# Supplementary material for: Developmental Strategy and Validation of the Midwifery Interventions Classification (MIC): A Delphi Study Protocol and Results from the Developmental Phase
Source: Healthcare (Basel). 2023 Mar 22;11(6):919. doi: 10.3390/healthcare11060919 (PMC10048446; doi:10.3390/healthcare11060919)
Supplement: Supplementary file 1 [file healthcare-11-00919-s001.zip › healthcare-2201410-supplementary.pdf]

Table S1. Overview of the data extraction for generating a preliminary MIC from the literature review

First author and year of publication	Geographic area	Study design	Objective	Category/domain	Class	Midwifery interventions
Alliman et al., 2019 [41]	USA	Descriptive analysis	To explore the medical and social risk factors, care processes, and quality outcomes of Medicaid beneficiaries in the AABC Strong Start sample compared with publicly reported US data in order to inform future research and Medicaid policy reform	Ancillary Services	//	Laboratory tests
						Social services and nutrition consultants
						Notification and referral for pediatric care
				Birth center/Primary care	//	Registration and orientation
						Informed consent
						History and physical
						Antepartum care
						Counseling/Education
						Continuous screening
						Admission to the birth center for labor
						Birth center
						Postpartum care
						Newborn care
						Education
						Discharge
						2-3 day home visit
						7-10 day office visit
						4-6 week exam
						Family planning
						Breastfeeding support
						Parenting support
				Hospital/Physician care	//	OB consultation
						Antepartum referral
						Intrapartum referral
						Hospital birth
						Postpartum or newborn referral
						Discharge

Blanc et al., 2016 [40]	Kenya	Observational study	To assess the validity of a large set of maternal and newborn health indicators that are included or have the potential to be included in population–based surveys	Initial client assesment	//	Woman referred to the facility because of a problem
						Provider washes hands with soap and water or uses antiseptic before initial examination
						Takes blood pressure
				Provide respectful care	//	Woman allowed to drink liquids or eat
						Encourages/assists woman to ambulate during labor
						Encourages/assists woman to assume different positions in labor
						Woman are allowed to have a support person during labor and delivery
						Support companion present during the birth
				First stage of labor	//	Induces labor with uterotonic
						Augments labor with uterotonic
						Uterotonics received (labor induction or augmentation)
						Membranes ruptured (labor induction or augmentation)
				Skilled birth attendance	//	Main provider labor nurse/midwife
						Main provider delivery doctor (ob/gyn)/medical resident
						Main provider delivery student nurse
				Second and third stage labor	//	Episiotomy performed
						Uterotonic received following delivery of the placenta
				Immediate postnatal newborn care	//	Baby is given to mother immediately after birth
						Baby placed skin-to-skin immediately on the mother
						Breastfeeding within the first hour of birth
						3 elements of essential newborn care (immediately dried, on mother's skin, breastfed within the first hour)
						3 elements of essential newborn care (immediately dried, 2 items on mother's skin, breastfeeding within the first hour)
						Low birthweight newborn (<2500g)
				Immediate postnatal care for the mother	//	Palpates uterus 15 min after delivery of the placenta
						First post–the delivery exam, the provider asks/ checks for bleeding
						First post–delivery exam, the provider examines the perineum

						First post–delivery exam, the provider takes the temperature
						First post–delivery exam, the provider takes blood pressure
						First post–delivery exam, the provider checks for involution
						Woman asked for pain relief medication during her stay
						Woman received pain relief medication
				Maternal outcomes	//	Cesarean section performed
						Reason for cesarean section - prolonged/obstructed labor
						Complications (bleeding, prolonged labor, none)
Butcher et al., 2020 [33]	USA	Observational study	Organizing nursing interventions and activities in a complete and systematic way	Basic physiology	Management of activity and physical exercise	Promotion of physical activity
					Management of elimination	Support in managing bowel function
						Management of urinary elimination
						Education on proper urinary habits
					Nutritional support	Nutrition
						Self-care assistance: nutrition
						Nutritional counseling
					Promotion of physical wellbeing	Pain management
						Massage
						Environmental Management: wellbeing
					Facilitation of self-care	Nutrition
						Self-care assistance
						Perineal care
						Self-care assistance: bathing/personal hygiene
				Complex physiological	Management of medications	Administration of analgesics
						Administration of medication

		Medication management
	Perioperative assistance	Intraoperative positioning
		Administration of anesthesia
		Post-anesthesia care
		Preoperative coordination
		Assistance during surgery
		Management of surgical instruments
		Preventive measures in case of surgery
		Preparation for surgery
		Regulation of intraoperative temperature
		Teaching: preoperative period
		Infection control: intraoperative phase
	Management of skin/wounds	Care of the incision site
		Suturing
		Wound care
	Thermoregulati on	Temperature regulation
	Management of tissue perfusion	Preventive measures for bleeding
		Reduction of bleeding
		Reduction of antepartum uterine bleeding
		Reduction of postpartum bleeding
		Capillary blood sampling
		Venous cannulation
		Intravenous therapy
		Blood sampling: venous blood sample
Behavior	Behavioral therapy	Support in behavior management
		Support in behavior modification
		Sharing of goals
		Contract with the patient
		Assistance in smoking cessation

		Prevention of substance abuse
	Cognitive therapy	Facilitation of learning
	Improvement of communication	Active listening<br>Improvement of socialization
	Coping assistance	Preventive guidance in critical situations<br>Improvement of coping<br>Counseling<br>Sexual counseling<br>Support in decision-making<br>Emotional support<br>Facilitation of grief processing: perinatal death<br>Presence<br>Improvement of safety<br>Enhancement of self-awareness<br>Improvement of self-efficacy<br>Improvement of self-esteem
	Patient education	Health education<br>Improvement of health literacy<br>Facilitation of learning<br>Improvement of readiness to learn<br>Parent education: child in the first year of life<br>Teaching: preoperative period<br>Teaching: nutrition of the child from 0 to 3 months of life<br>Teaching: safety of the child from 0 to 3 months of life<br>Family planning: contraception
	Promotion of psychological wellbeing	Guided visualization
Safety	Crisis management	Management of severity codes<br>Emergency assistance

		First aid
		Triage: emergency center
		Telephone triage
	Risk management	Allergy management
		Environment management
		Environment management: safety
		Prevention of falls
		Screening
		Vaccination management
		Latex use precautions
		Infection control
		Identification of the assisted person
		Surveillance
		Monitoring of vital parameters
Family	Pregnancy assistance	Assistance to the person: pregnancy
		Reduction of antepartum uterine bleeding
		Reduction of postpartum bleeding
		Facilitation of grief processing: perinatal death
		Identification of risks: family expecting a child
		Surveillance: post-term pregnancy
		Assistance during childbirth
		Preparation for childbirth
		Electronic fetal monitoring: antepartum
		Electronic fetal monitoring: intrapartum
		Family planning: contraception
		Assistance to the child: newborn
		Intrapartum assistance
		Intrapartum assistance: high-risk childbirth
		Labor induction
		Labor suppression

		Lactation suppression
		Non-nutritive sucking
		Phototherapy: newborn
		Postpartum and puerperium assistance
		Prenatal assistance
		Resuscitation: fetus
		Resuscitation: newborn
		Obstetric ultrasound
		Promotion of the integrity of the family expecting a child
	Assistance in child growth	Bottle feeding
		Breastfeeding counseling
		Parent education: child in the first year of life
		Teaching: nutrition of the child from 0 to 3 months of life
		Teaching: safety of the child from 0 to 3 months of life
		Promotion of parent-child attachment
		Cup feeding: newborn
		Promotion of parenting role
	Assistance throughout life	Empowerment of the role
		Support to the caregiver
		Promotion of family involvement
		Support to the family
Healthcare system	Sustaining adaptation with the healthcare system	Preoperative coordination
		Support to the decision-making process
		Welcome to a healthcare or social facility
		Case management
		Cultural mediation
		Guide to the healthcare system
		Protection of the rights of the assisted person
		Discharge planning
		Facilitation of visits

Management of the healthcare system	Emergency cart control<br>Interpretation of laboratory data<br>Peer review<br>Collaboration with the doctor<br>Improvement of collaboration<br>Tutoring: Staff<br>Tutoring: Students<br>Evaluation of equipment<br>Quality monitoring<br>Management of laboratory samples<br>Staff supervision<br>Management of supplies<br>Staff development<br>Management of technology<br>Transfer to another facility<br>Transfer within the facility<br>Management of economic resources
Management of information	Surveillance: teleassistance<br>Consultancy<br>Documentation<br>Collection of forensic data<br>Exchange of health-related information<br>Monitoring of healthcare policy<br>Incident reporting - report on an accidental event<br>Multidisciplinary care consultancy<br>Prescription: diagnostic test<br>Prescription: non-pharmacological treatment<br>Data collection for research purposes<br>Shift change communications<br>Telephone consultation

						Telephone follow-up
				Community	Promotion of community health	Case management<br>Community health development<br>Management of economic resources<br>Health education<br>Monitoring of healthcare policy<br>Management of vaccinations<br>Program development
					Management of community risks	Screening<br>Risk identification.
Day et al., 2021 [32]	Bangladesh, Nepal, and Tanzania	Observational study	To assess the validity of indicators of maternal and newborn healthcare coverage around the time of birth in survey data and routine facility register data	Labor and delivery ward	//	Uterotonics to prevent postpartum hemorrhage<br>Early initiation of breastfeeding<br>Neonatal resuscitation by bag-mask ventilation
				Kangaroo mother care ward or corner	//	Skin-to-skin contact or kangaroo mother care position between mother and baby
				Newborn ward or pediatrics ward	//	Antibiotic treatment for neonatal infection
Devane et al., 2019 [39]	Ireland	Observational study	To report on the development and prioritization of a national suite of Quality Care Metrics (QCM), and their associated indicators, for midwifery care processes in Ireland	//	//	Midwifery Plan of Care<br>Booking<br>Abdominal examination (after 24 weeks gestation) on current or last assessment<br>Intrapartum fetal wellbeing<br>Intrapartum fetal wellbeing cardiotocography (CTG)<br>Intrapartum maternal wellbeing<br>Risk assessment for venous thromboembolism (VTE) in pregnancy and the puerperium<br>Immediate post-birth care<br>Communication (Clinical Midwifery Handover)<br>Pain management (other than labor)<br>Infant feeding

						Postnatal care (daily midwifery care processes)<br>Post-birth discharge planning for going home<br>Medication administration<br>Medication, storage, and custody (excluding MDSs)<br>MDA drugs<br>Intravenous fluid therapy<br>Clinical Record Keeping
Diamond-Smith et al., 2022 [31]	Africa	Literature review	To summarize and describe respectful maternal care-promoting interventions during childbirth implemented in Africa	Supply-side domains	//	Train healthcare personnel<br>Improve facility environment<br>Implement quality improvement programs<br>Enact policies or guidelines<br>Support provider wellness<br>Address other drivers of RMC
				Demand-side domains	//	Advocate for respectful maternal care<br>Educate women giving birth<br>Engage community members
				Combination of both supply and demand side domains	//	Implement social accountability mechanisms
Escuriet et al., 2015 [38]	Spain	Literature review	To provide a critical literature review on the tools and indicators currently being used to measure the performance of maternity care within Europe and to identify the dominant focus of existing measurements	Process	//	Antenatal care<br>Intrapartum care<br>Postnatal care<br>Neonatal care
Flenady et al., 2016 [30]	Australia	Observational study	To assess the status of global indicators by describing the currently available data to address the WHO Essential Interventions and to develop a robust suite of indicators	Preconception interventions	//	Family planning (preconception advice and counseling about the choice of contraception)<br>Prevention and Management of STIs, including HIV for PMTCT of HIV and syphilis<br>Folic acid fortification and/or supplementation to prevent neural tube defects

and respective data points to monitor WHO Essential Interventions – both screening and management components – for use in eRegistries.	Antenatal care interventions	//	Antenatal care essential package (four-visit schedule)
			Iron and folic acid supplementation during pregnancy
			Tetanus immunization in pregnancy for preventing neonatal tetanus
			Prophylactic antimalarial for preventing malaria in pregnancy
			Insecticide Treated Bednets (ITBN) for preventing malaria in pregnancy
			Smoking cessation during pregnancy
			Screening and treatment of syphilis (during pregnancy)
			Prevention and Management of HIV and prevention of mother-to-child-transmission (PMTCT) in pregnancy
			Calcium supplementation in pregnancy for preventing pre-eclampsia
			Low-dose aspirin for preventing pre-eclampsia in high-risk women
			Antihypertensive drugs for treating severe hypertension in pregnancy
			Magnesium sulfate to prevent and treat eclampsia
			External Cephalic Version (>36 weeks) to reduce malpresentation at term
			Induction of labor for Management of prelabour rupture of membranes (PROM) at term
			Antibiotics for Management of preterm prelabour rupture of membranes (pPROM)
			Corticosteroids for prevention of neonatal respiratory distress syndrome (RDS) in preterm labor
			Safe abortion for the Management of unintended pregnancy
	Interventions for care at birth	//	Social support during childbirth
			Prophylactic antibiotic for cesarean section
			Cesarean section for maternal/fetal indication (e.g., obstructed labor and central placenta previa)
			Prophylactic uterotonic/active Management of the third stage to prevent postpartum hemorrhage
			Induction of labor for prolonged pregnancy
			Uterotonics for Management of postpartum hemorrhage

						Manual removal of placenta (only by professional health workers) for Management of postpartum hemorrhage<br>Initiation or continuation of HIV therapy for HIV-positive women
				Interventions for postpartum care (of the mother)	//	Advice and provision of family planning<br>Prevent, measure, and treat maternal anaemia<br>Detection and Management of postpartum sepsis<br>Screening and initiation or continuation of antiretroviral therapy for HIV
				Interventions for newborn care	//	Promotion and provision of thermal care for all newborns to prevent hypothermia<br>Promotion and support for early initiation and exclusive breastfeeding (within the first hour)<br>Promotion and provision of hygienic cord and skin care<br>Neonatal resuscitation with a bag and mask for babies who do not breathe at birth<br>Newborn immunization<br>Presumptive antibiotic therapy for newborns at risk of bacterial infection<br>Case management of neonatal sepsis, meningitis, and pneumonia<br>Initiation of ART in babies born to HIV-infected mothers
				Interventions for small and ill babies	//	Kangaroo mother care (KMC) for preterm and for <2000g babies<br>Extra support for feeding the small and preterm baby<br>Prophylactic and therapeutic use of surfactant to prevent respiratory distress syndrome in preterm babies<br>Continuous positive airway pressure (CPAP) to manage preterm babies with respiratory distress syndrome<br>Management of newborns with jaundice
Lavender, 2016 [37]	UK	Narrative review	To synthesize the existing quality frameworks in the context of evidence-based interventions for essential care.	Childbirth	//	Induction of labor for prolonged pregnancy<br>Prophylactic uterotonics to prevent postpartum hemorrhage<br>Active Management of the third stage of labor to prevent postpartum hemorrhage<br>Management of postpartum hemorrhage (e.g., uterotonics, uterine massage)

						Cesarean section for maternal/fetal indication
						Prophylactic antibiotics for cesarean section
				Postnatal (mother)	//	Family planning
						Prevent and treat maternal anemia
						Detect and manage postpartum sepsis
						Screen and initiate or continue antiretroviral therapy for HIV
				Postnatal (newborn)	//	Immediate thermal care (newborn)
						Initiation of exclusive breastfeeding (within the first hour)
						Hygienic cord and skin care
						Neonatal resuscitation with bag and mask (professional health worker)
						Case management of neonatal sepsis, meningitis, and pneumonia
						Kangaroo mother care for preterm and for less than 2000g babies
						Management of newborn with jaundice
						Surfactant to prevent respiratory distress syndrome in preterm babies
						Continuous positive airway pressure (CPAP) to manage babies with respiratory distress syndrome
						Extra support for feeding small and preterm babies
						Presumptive antibiotic therapy for newborns at risk of bacterial infections
Lazzaretto et al., 2018 [2]	Italy	Literature review	To identify the recommended intrapartum care indicators in order to measure the quality of midwifery care.	General midwifery procedures	//	Coping with pain
						Intrapartum Interventions
						Free maternal position
						Cesarean section
						Preterm delivery
						Intrapartum nutrition
						FHR monitoring
						Term newborn admitted to NICU
						Birth companion in labor
						Free maternal position

						Maternal wellbeing<br>Partogram<br>One-to-one midwifery care<br>Place of birth<br>TOLAC<br>Spontaneous delivery<br>Timing of birth<br>Cord clamp<br>Preterm newborn - NICU
				Midwifery procedures in the I stage	//	Interventions I stage<br>Free maternal position<br>One-to-one midwifery care
				Midwifery procedures in the II stage	//	Episiotomy<br>Interventions II stage<br>Expulsive contractions<br>Free maternal position
				Midwifery procedures in the III stage	//	Interventions III stage<br>Cord clamp
						Skin to skin
				Midwifery procedures in the IV stage	//	Perineum<br>Breastfeeding<br>Postpartum hemorrhage<br>Suturing<br>Interventions IV stage<br>Vaginal examination
Nababan et al., 2017 [29]	Bangladesh	Observational study	To examine the effectiveness of the SCC in improving childbirth practices at a district hospital in Bangladesh	Childbirth practices	On admission	Partograph use<br>Appropriate maternal infection management<br>Appropriate eclampsia management<br>Hand hygiene<br>Birth companion present

						Intrapartum counseling
					Just before pushing	Emergency birth helper was identified
						Appropriate maternal infection management
						Appropriate eclampsia management
						Supplies for mothers and babies
					Soon after birth (within 1 h)	Active Management of the third stage of labor
						Appropriate newborn thermal and resuscitation
						Maternal blood loss assessment and treatment
						Appropriate maternal infection management
						Appropriate eclampsia management
						Newborn referral
						Appropriate newborn infection management
						Newborn special care
						Breastfeeding and skin-to-skin contact
						Postpartum counseling
					Before discharge	Maternal blood loss assessment
						Appropriate maternal infection management
						Appropriate newborn infection management
						Newborn feeding assessment
						Family planning counseling
						Discharge counseling
						Follow-up visit counseling
Obara et al., 2014 [28]	WHO Western Pacific Region	Observational study	WHO and the UNICEF Regional Action Plan for Healthy Newborn Infants provide a platform for countries to scale up Early Essential Newborn Care (EENC). The plan emphasizes the creation of an enabling environment for the practice of EENC;	Intrapartum care	All	Labour Monitoring (Partograph)
						Intrapartum care
					Preterm and low birth weight	Elimination of unnecessary inductions and C-sections
						Antenatal steroids
						Antibiotics for preterm PROM
					Sick newborns	Assisted delivery
						C-section
				Newborn care	All	Drying

			thereby preventing 50.000 newborn deaths annually			Skin-to-skin contact<br>Clamping and cutting the cord appropriately<br>Initiating exclusive breastfeeding<br>Routine care- eye care, Vitamin K, immunizations, weighing and examinations
					Preterm and low birth weight	Kangaroo mother care<br>Breastfeeding support<br>Immediate treatment of suspected infection
					Sick newborns	Resuscitation<br>Antibiotic treatment
Pricilla et al., 2017 [27]	India	Observational study	To describe the quality of the antenatal care provided by nurse midwives of an urban health center concerning low-risk mothers and to document the maternal and early neonatal outcomes of the enrolled mothers during the period of study.	Interpersonal interaction	//	Greetings<br>Politeness<br>Addressing concerns<br>Answering concerns<br>Explaining before examination
				Physical examination	//	Gestational age calculated<br>Height measured<br>Weight measured<br>Blood pressure checked<br>Signs of anemia checked<br>Presence of edema examined<br>Fundal height measured<br>Metro gram marked<br>Presentation checked<br>Fetal heart checked
				Diagnostic tests	//	Urine albumin/sugar<br>Haemoglobin estimated<br>HIV tested
				Prophylactic drugs	//	Tetanus toxoid injection given<br>Iron and folic acid take

				Health education	//	Diet advice on Iron rich food
						Advice on preparedness for delivery
						Information about danger signs (maternal and foetal)
						Breast/nipple care advice
						Family planning advice
Saturno-Hernàndez et al., 2019 [26]	Spain	Systematic review	To systematically review indicators for the central phases of the maternal and child healthcare continuum of care (pregnancy, childbirth, newborn care and postpartum).	Structure	//	Design, policy, and health system context
						Human resources and infrastructure
						Mother, child, family and community context
				Process	//	Preventive activities
						Diagnosis and screening
						Treatment of pathologic conditions
						Other maternal and childcare processes
				Outcome	//	Maternal mortality
						Child mortality
						Maternal morbidity
						Child morbidity
						Safety in the care of the mother
						Safety in childcare
						Satisfaction
						Other health outcomes
				Health determinants	//	Health knowledge and a healthy lifestyle
						Working and living conditions
						Environmental and geographic factors
						Demographics and other health statistics
Simpson et al., 2019 [35]	USA	Observational study	To describe the development of an adaptation of the MISSCARE Survey to measure missed nursing care in the perinatal setting.	//	//	Assist women to the shower or tub for hydrotherapy
						Assess the effectiveness of medications
						Check bladder status/voiding needs every 2 hours
						Assess pain status every hour
						A thorough review of prenatal records

						Patient teaching about procedures, tests, and other diagnostic studies<br>Medications administered within 30 minutes before or after the scheduled time<br>Administer oxytocin as per orders or protocol
						Response to call light is initiated within 5 minutes
						Emotional support to patient and/or family
						Patient bathing, skincare, or pedicure
						Recognize and respond to indeterminate (Category II) or abnormal (Category III) FHR changes within 15 minutes<br>Recognize and respond to excessive uterine activity within 20 minutes<br>Recognize and respond to new maternal complications within 10 min (e.g.hypertension, bleeding)<br>Focused reassessments according to patient condition (e.g., FHR and uterine activity assessment q 15 minutes for women receiving oxytocin for labor induction or augmentation)<br>Real-time or near real-time documentation of maternal-fetal assessments during labor, birth, and recovery<br>Patient teaching about signs and symptoms, when to call after discharge from OB triage<br>Monitor intake and output
						Provide skin-to-skin mother-baby care immediately after birth
						Assess vital signs as ordered or per protocol
						Provide thorough patient handoff
						Hand washing
						Breastfeeding within 1 hour after birth for women who are breastfeeding<br>Recovery care consisting of 2 hours of every 15-minute maternal assessment and 2 hours of every 30-minute newborn assessments<br>Notification of physician or nurse-midwife in a timely manner to attend the birth
Ueda et al., 2019 [34]	Japan	Observational study	To update the quality indicators developed for low-risk labor care in Japan	Pregnancy period	//	Primipara who has enrolled in a childbirth class about antenatal care and delivery by 36 weeks gestation<br>Discussed a birth plan

in 2012, mainly drawing on new or updated clinical practice guidelines and making the process clearly visible and assessable	Intrapartum period	//	Initial assessment of labor risk at admission
			Assessment during the first stage of labor
			Assessment during the second stage of labor
			Women with a term, singleton infant in vertex position delivered by cesarean section
			Women with a term, singleton infant in vertex position delivered by vaginal delivery
			Women with a term, singleton infant in vertex position delivered by instrumental delivery
			Women with a term, singleton infant in vertex position delivered by labor induction
			Term infants with Apgar score less than 7 at five minutes after birth
			Living infant with birth injuries
			Respiratory support: resuscitation for asphyxiated term neonate with low oxygen concentrations and oxygen saturation measured by pulse oximetry immediately after birth
			Women with perineal tears and no perineorraphy
			Second-degree perineal laceration
			Third fourth-degree perineal laceration
			Postpartum hemorrhage of more than 500g within 2 hours of birth
	Within 1 week after childbirth	//	Infant admission to pediatrics department within 1 week after birth (excludes those with congenital anomalies)
			Infants that were fed only breast milk at the time of discharge from the hospital
			Peer review of severe adverse events with medical staff
			The woman switched to receive care provided primarily by obstetricians from the midwifery ward
			Mother received cessation counseling intervention (including guidance on smoking cessation) if identified as either a tobacco user or passive smoker
	Within 1 month after birth	//	Infant administered vitamin K three times by one month after birth
			Infants who had been fed only breast milk at the time of the health examination for children of 1 month of age

New indicators	//	Women receiving antibiotic prophylaxis during childbirth if maternal group B Streptococcus infections are identified at 33 to 37 weeks' gestation<br>Infants are offered the necessary resuscitation in the first minutes after birth, evaluating their condition in line with the Japanese Neonatal Resuscitation Algorithm<br>Women receiving uterotonics for the prevention of postpartum hemorrhage during the third stage of labor<br>Women having early skin-to-skin contact with their babies if they wish, soon after birth in secure surroundings<br>Women planning spontaneous vaginal birth in a midwifery ward and being able to follow that plan<br>Infants are given formula supplementation without medical rationale from birth to discharge in term infants, even though the mother intended to breastfeed<br>Women having a fall during their hospitalization<br>Women and infants readmitted within 30 days of discharge<br>Women being screened for antenatal or postnatal depression using a validated questionnaire<br>Women and infants have complete medical records based on all quality indicators<br>Women have a review of their childbirth experience and support from the midwives and other staff who assisted at the birth.<br>Women have been encouraged and supported to adopt the most comfortable positions throughout the second stage of labor
